# Effects of Organic and Inorganic Nitrogen on the Growth and Production of Domoic Acid by *Pseudo-nitzschia multiseries* and *P. australis* (Bacillariophyceae) in Culture

**DOI:** 10.3390/md13127055

**Published:** 2015-11-26

**Authors:** Véronique Martin-Jézéquel, Guillaume Calu, Leo Candela, Zouher Amzil, Thierry Jauffrais, Véronique Séchet, Pierre Weigel

**Affiliations:** 1 FR CNRS-Université de Nantes-Ifremer 3473, EA 2160, Pôle Mer et Littoral, 2 rue de la Houssinière, BP 92208, 44322 Nantes Cédex 3, France; calu.guillaume@wanadoo.fr (G.C.); leocandela09@gmail.com (L.C.); thierry.jauffrais@univ-angers.fr (T.J.); 2 CNRS UMR 7266, LIENSs, Université La Rochelle, 2 rue Olympe de Gouges, 17000 La Rochelle, France; 3 Laboratoire Phycotoxines, IFREMER, rue de l’Ile d’Yeu, BP 21105, 44311 Nantes, France; zouher.amzil@ifremer.fr (Z.A.); veronique.sechet@ifremer.fr (V.S.); 4 CNRS-Université d’Angers, UMR 6112-LPG-BIAF, 2 boulevard Lavoisier, 49045 Angers, France; 5 CNRS-Université de Nantes, UMR 6204-U3B, 2 rue de la Houssinière, BP 92208, 44322 Nantes Cédex 3, France; pierre.weigel@univ-nantes.fr

**Keywords:** *Pseudo-nitzschia*, toxic diatoms, nitrogen, amino acids, domoic acid

## Abstract

Over the last century, human activities have altered the global nitrogen cycle, and anthropogenic inputs of both inorganic and organic nitrogen species have increased around the world, causing significant changes to the functioning of aquatic ecosystems. The increasing frequency of *Pseudo-nitzschia* spp. in estuarine and coastal waters reinforces the need to understand better the environmental control of its growth and domoic acid (DA) production. Here, we document *Pseudo-nitzschia* spp. growth and toxicity on a large set of inorganic and organic nitrogen (nitrate, ammonium, urea, glutamate, glutamine, arginine and taurine). Our study focused on two species isolated from European coastal waters: *P. multiseries* CCL70 and *P. australis* PNC1. The nitrogen sources induced broad differences between the two species with respect to growth rate, biomass and cellular DA, but no specific variation could be attributed to any of the inorganic or organic nitrogen substrates. Enrichment with ammonium resulted in an enhanced growth rate and cell yield, whereas glutamate did not support the growth of *P. multiseries*. Arginine, glutamine and taurine enabled good growth of *P. australis*, but without toxin production. The highest DA content was produced when *P. multiseries* grew with urea and *P. australis* grew with glutamate. For both species, growth rate was not correlated with DA content but more toxin was produced when the nitrogen source could not sustain a high biomass. A significant negative correlation was found between cell biomass and DA content in *P. australis*. This study shows that *Pseudo-nitzschia* can readily utilize organic nitrogen in the form of amino acids, and confirms that both inorganic and organic nitrogen affect growth and DA production. Our results contribute to our understanding of the ecophysiology of *Pseudo-nitzschia* spp. and may help to predict toxic events in the natural environment.

## 1. Introduction

Nitrogen is one of the most abundant elements in aquatic systems and an essential component for living biomass. Nitrogen availability in the environment is a major cue that stimulates the growth and activity of primary producers [1,2]. Over the last century, human activities have altered the global nitrogen cycle, and anthropogenic inputs of both inorganic and organic nitrogen species have increased around the world, causing significant changes to the functioning of estuarine and coastal ecosystems. Environmental concerns about eutrophication focus on the most common nitrogen forms in aquatic ecosystems, *i.e.*, the inorganic ions, nitrate and ammonium. As fertilizers, these nitrogen sources stimulate the production of biomass, and pollution related to them aggravates the proliferation of algae. Organic nitrogen from sewage also influences the development and maintenance of microalgae. Dissolved organic nitrogen (DON) often accounts for the majority of total nitrogen in the environment, while organic compounds such as urea and amino acids have been measured in most coastal systems [3]. Today, organic compounds contribute >50% of the total nitrogen fertilizer [4] and the use of urea has increased 100-fold in the past four decades [5]. A significant impact of this nitrogen loading is the worldwide increase in harmful algal blooms in coastal waters [6,7,8,9].

Nitrogen enrichment is thus likely to cause not only an increased biomass but also a change in algal species composition. Of particular concern is the rise in toxigenic phytoplankton, especially diatoms of the genus *Pseudo-nitzschia* that produce the neurotoxin domoic acid (DA) [10]. Clinical symptoms associated with DA intoxication are known as Amnesic Shellfish Poisoning (ASP) [10]. To date, 19 species of this genus have been confirmed as producing DA [10,11,12,13,14,15,16,17]. One other diatom is reported to be toxigenic and to produce DA, *Nitzschia navis-varingica* [18]. Despite the widespread interest in *Pseudo-nitzschia* spp. ecology and the geographical extent of its monitoring [19], environmental cues that trigger the toxicity of this diatom are still a matter of debate [7,10,11,20]. Interpretation of *Pseudo-nitzschia* spp. ecophysiology in the field has been mostly related to major nutrients (N-NO_3_, P-PO_4_ and Si-Si(OH)_4_) [21]. It is recognized that *Pseudo-nitzschia* growth and DA production benefit from nitrate loading in the environment [7,10,11,21,22,23]. Moreover, there is evidence that nitrogen enrichment and silicon limitation in the environment favor DA production [21]. Because diatoms can assimilate both inorganic and organic nitrogen [3,24], it is not surprising that *Pseudo-nitzschia* spp. can benefit from these sources for growth and even toxin production [7,11]. To date, growth and toxicity of *Pseudo-nitzschia* spp. in response to inorganic or organic nitrogen sources appear quite diverse. Impacts of inorganic sources, such as nitrate and ammonium, on growth and DA production were first explored in cultures of *P. multiseries* [25,26,27]. Evidence that the inorganic sources may influence both the growth rate and toxin production of other *Pseudo-nitzschia* species was subsequently provided [10,11,28,29]. In contrast, organic nitrogen species have been less widely tested. In particular, most of the studies have not addressed nitrogen supplied as amino acids. The first report concerned *P. multiseries* and dealt only with growth on glutamine or urea as organic nitrogen sources [30]. The effects of DON on DA production are only now beginning to be known [7,29,31,32]. Studies on the toxin response of urea have been carried out on laboratory cultures of *P. australis* [31], *P. multiseries* [28,33], *P. calliantha*, *P. fraudulenta* [28], *P. cuspidata*, *P. fryxelliana* [29] and *P. pungens* [33]. Each of these studies has shown broad intra- and inter-specific variations in growth and DA production, depending in the nitrogen source tested. The cosmopolitan nature of toxigenic *Pseudo-nitzschia* species reinforces the need to understand better the environmental control of growth and DA production.

The purpose of the present study is to provide new information about the influence of nitrogen sources on two toxic species of *Pseudo-nitzschia*. Here, we describe the effects of inorganic and organic nitrogen on the growth and toxin production of two species, *P. multiseries* CCL70 and *P. australis* PNC1, isolated from European coastal waters (Western Brittany and the Thames estuary, respectively). These *Pseudo-nitzschia* species were tested on a broad spectrum of nitrogen sources: inorganic (nitrate and ammonium) and organic (urea, glutamate, glutamine, arginine and taurine). Nitrate, urea and ammonium are known as major nitrogenous nutrients for marine phytoplankton. Glutamate and glutamine are major metabolites in nitrogen nutrition. Arginine is degraded to urea and ornithine during the urea cycle and these amino acids are confirmed nitrogen sources for diatoms [3]. Taurine is one of the major amino acids measured in invertebrates and fishes [34,35] and can be released into seawater. In particular, it was the major amino acid excreted by *Crassostrea gigas* housed in a system with natural seawater [36]. Taurine can be used as a nitrogen source by *Nitzschia* sp. [37] and it is also a possible osmotic regulator in *P. multiseries* [38]. Our study aims to reveal new aspects of *Pseudo-nitzschia* nitrogen metabolism and to help decipher the regulation of DA production.

## 2. Results

### 2.1. Growth

Nitrogen substrates influenced the specific growth rate, the onset of the stationary phase and the maximum cell abundance of *P. multiseries* CCL70 and *P. australis* PNC1. However, the results are different for the two species (Table 1).

**Table 1:** Specific growth rate, mean biomass in stationary phase, mean cellular domoic acid (DA), for *P. multiseries* CCL70 and *P. australis* PNC1 grown on nitrate, ammonium, urea, arginine, glutamine, glutamate and taurine (the latter for *P. australis* only); nd = not detectable; *n* = 2 ± SE.

	*P. multiseries* CCL70			*P. australis* PNC1		
	Specific Growth Rate µ (day^−1^)	Mean Biomass in Stationary Phase (10^3^ Cells mL^−1^)	Mean DA (pg·Cell^−1^)	Specific Growth Rate µ (day^−1^)	Mean Biomass in Stationary Phase (10^3^ Cells mL^−1^)	Mean DA (fg·Cell^−1^)
Nitrate	0.80 ± 0.32	109.0 ± 32.0	1.12 ± 0.23	0.48 ± 0.003	336.0 ± 3.2	14.5 ± 0.1
Urea	0.60 ± 0.01	123.0 ± 4.1	1.62 ± 0.09	0.44 ± 0.01	336.0 ± 6.8	10.9 ± 0.5
Ammonium	1.12 ± 0.12	161.0 ± 6.5	0.17 ± 0.02	0.56 ± 0.003	191.0 ± 2.6	22.7 ± 2.4
Arginine	0.63 ± 0.01	139.0 ± 2.4	0.49 ± 0.01	0.47 ± 0.02	360.0 ± 9.4	nd
Glutamine	0.59 ± 0.01	114.0 ± 6.3	0.22 ± 0.02	0.60 ± 0.01	157.0 ± 3.5	nd
Glutamate	0.08 ± 0.03	14.0 ± 3.7	nd	0.37 ± 0.03	136.0 ± 28.0	30.3 ± 3.9
Taurine	-	-	-	0.33 ± 0.02	365.0 ± 25.0	nd

**Figure 1:**
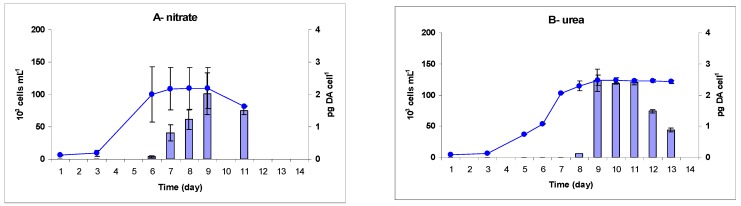

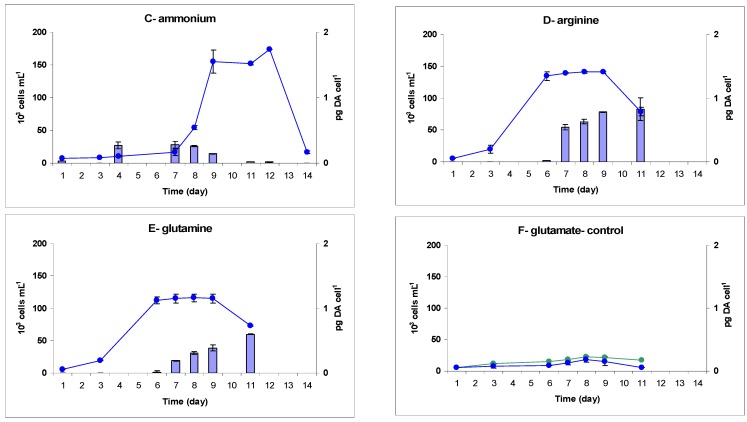
Cell abundance (●) and cellular domoic acid (blue bars) over time in *Pseudo-nitzschia multiseries* CCL70 grown on nitrate (**A**); urea (**B**); ammonium (**C**); arginine (**D**); glutamine (**E**) and glutamate (**F**). *n* = 2 ± SE. No domoic acid was detected in growth with glutamate; growth of the control culture is shown instead in (**F)** (●). Note different scale on *Y*-axis for DA content in panels (**A**,**B**) and (**C**–**F**).

Growth of *P. multiseries* was characterized by a short lag phase (Figure 1), with the exception of the population grown with ammonium, for which the exponential phase started later. Time of onset of the stationary phase was similar for nitrate, arginine and glutamine (day 6; Figure 1), whereas the exponential phase stopped later for ammonium and urea (day 9; Figure 1). However, the stationary phase was shorter for nitrogen sources other than urea, which resulted in a cell decline after three days (Figure 1A–E). *P. multiseries* exhibited a different growth response with glutamate. This amino acid did not sustain good growth and a very low growth rate was obtained (0.08 day^−1^) (Table 1, Figure 1F). In contrast, the highest specific growth rates occurred in cultures using ammonium (1.12 day^−1^) and nitrate (0.80 day^−1^) (Table 1). Growth rates were lower and not substantially different for cultures grown on arginine, glutamine or urea, with values ranging from 0.59 to 0.63 day^−1^ (Table 1). Cell abundance in the stationary phase showed similar patterns with respect to the nitrogen source (Table 1). The highest biomass was produced with ammonium (161 × 10^3^ cells mL^−1^, Table 1). Abundances were lower in cultures grown with nitrate, urea, glutamine and arginine with values ranging from 109 to 139 × 10^3^ cells mL^−1^, Table 1) In accordance with the low growth rate measured with glutamate, a lower biomass was produced with this amino acid (14 × 10^3^ cells mL^−1^, Table 1) and growth was not substantially different from the culture without added nitrogen (Figure 1F).

**Figure 2:**
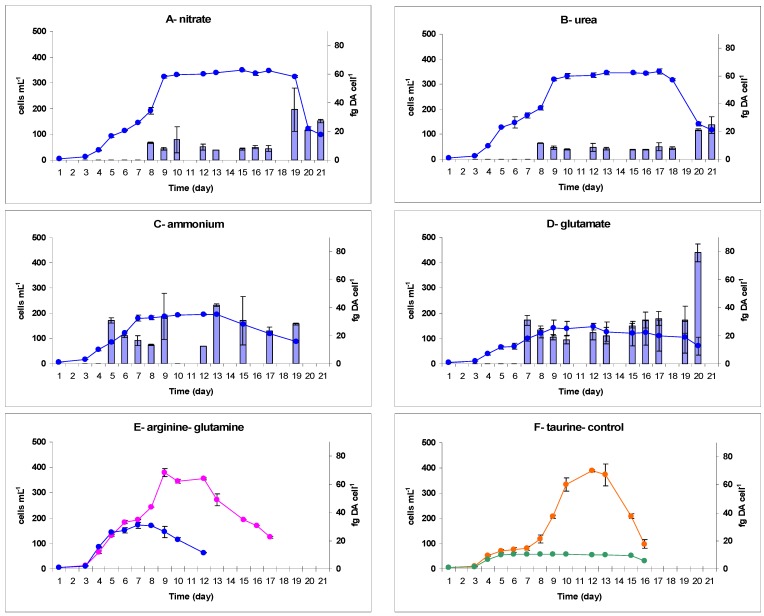
Cell abundance (●) and cellular domoic acid (blue bars) over time in *Pseudo-nitzschia australis* PNC1 grown on nitrate (**A**); urea (**B**); ammonium (**C**); glutamate (**D**); arginine (**E**); glutamine (**E**) and taurine (**F**). *n* = 2 ± SE. No domoic acid was detected in growth with arginine (**E** ●), glutamine (**E** ●) and taurine (**F** ●); growth of the control culture is shown instead in (**F** ●).

*P. australis* (PNC1) was cultured with taurine in addition to the above nitrogen species tested on *P. multiseries*, and all seven nitrogen conditions allowed growth (Table 1). Specific growth rates were subtanstially higher with glutamine (0.60 day^−1^) and ammonium (0.56 day^−1^) than with nitrate, urea or arginine (0.44 to 0.48 day^−1^). Glutamate and taurine exhibited the lowest growth rate (0.37 day^−1^ and 0.33 day^−1^, respectively). High biomasses were produced with taurine and arginine (365 × 10^3^ and 360 × 10^3^ cells mL^−1^, Table 1), and nitrate and urea (336 × 10^3^ cells mL^−1^, Table 1), whereas cellular abundances were substantially lower when grown on ammonium, glutamine and glutamate (191 × 10^3^, 157 × 10^3^ and 136 × 10^3^ cells mL^−1^, respectively) (Table 1). The highest growth rates were therefore not necessarily associated with the highest biomass (Table 1). Time of onset of the stationary phase for *P. australis* did not fit the same pattern for all nitrogen conditions. Growth on glutamine and ammonium stopped earlier (days 5 and 7, respectively) than on arginine (day 8), nitrate, urea, glutamate (day 9) and taurine (day 10) (Figure 2).

Analysis of variance (ANOVA) determined that nitrogen sources, *Pseudo-nitzschia* species, and the interaction of both significantly affected specific growth rate (*p* < 0.005) and mean or maximum cellular abundances (*p* < 0.001). *P. multiseries* and *P. australis* showed different nutritional preferences and growth responses to nitrogen sources. No patterns were found that were common to both species, neither for their specific growth rate nor for their cellular abundance in stationary phase. *P. multiseries* exhibited the highest growth rates and *P. australis* produced the highest biomasses (Table 1). Growth of *P. multiseries* was characterized by a consistent co-evolution of its kinetic parameters in all nitrogen treatments. This was revealed by the significant positive correlation between biomass and growth rate (*p* < 0.01; *R*^2^ = 0.88; Spearman test: ρ = 0.964; *p* < 0.001). In contrast, the effects of each nutrient condition on the growth parameters of *P. australis* appeared more variable. There was no positive relationship between growth rate and maximum biomass. Instead, a negative but not significant tendency was observed between the two parameters (*p* = 0.67; *R*^2^ = 0.03, Spearman test: ρ = 0.04; *p* = 0.885).

### 2.2. Toxicity

*P. multiseries* produced DA under all nitrogen conditions except for glutamate (Figure 1, Table 1). It is, however, notable that DA was not detected in the culture medium during our monitoring (Table S1). This species produced the highest amount of toxin in the presence of urea (1.62 pg·cell^−1^). For cells growing on nitrate, arginine and glutamine, the DA levels were 1.12, 0.49 and 0.22 pg·cell^−1^, respectively. The lowest cellular DA was found with ammonium (0.17 pg·cell^−1^, Table 1).

Toxin production by *P. australis* did not follow the same trend as did *P. multiseries*. Despite good growth, no cellular or dissolved DA was detected in *P. australis* cultured with glutamine, arginine or taurine although dissolved DA was found in all the other nitrogen conditions (Figure 2, Table 1, Table S1). Moreover, cellular DA levels in this species were two to three orders of magnitude lower than in *P. multiseries*. The highest DA contents were found during growth with glutamate (30.3 fg·cell^−1^) and ammonium (22.7 fg·cell^−1^); cultures grown with nitrate and urea produced substantially less toxin (14.5 and 10.8 fg·cell^−1^, respectively) (Table 1).

For both species, the highest DA content was measured during the stationary phase in most of the nitrogen conditions tested (nitrate, urea and amino acids) (Table S1, Figure 1 and Figure 2). Nevertheless, regarding the nitrogen substrates, *P. multiseries* and *P. australis* exhibited different patterns for the change in cellular DA during this phase of growth. The dynamics of DA production by *P. multiseries* were similar with nitrate, arginine and glutamine. With these three substrates, toxin accumulated during the stationary phase, reaching the maximum concentration only in the late stationary or senescence phases (Figure 1A,D,E). Conversely, *P. multiseries* growing with urea produced maximum levels of toxin at the mid-stationary phase, which then decreased during the late-stationary phase (Figure 1B). In contrast, DA production started during the mid-exponential phase in all cultures of *P. australis* (Figure 2). Cellular DA was present until the end of growth and increased in the late-stationary phase for cultures grown with nitrate, urea and glutamate (Figure 2A,B,D). The toxicity pattern of both species was different with the substrate ammonium compared to the other nitrogen treatments. Cellular DA was detected earlier in the exponential growth phase (Figure 1C and Figure 2C, Table S1). However, the pattern for each species was different during growth. *P. multiseries* produced the highest DA concentration during a prolonged lag phase and into the beginning of the exponential growth phase, whereas the DA content decreased by almost 40% during the early-stationary phase and was thereafter only at very low concentrations (Figure 1C). In contrast, *P. australis* started to produce toxin during the mid-exponential phase and, like the other N sources, continued during the stationary phase (Figure 2C).

**Figure 3:**
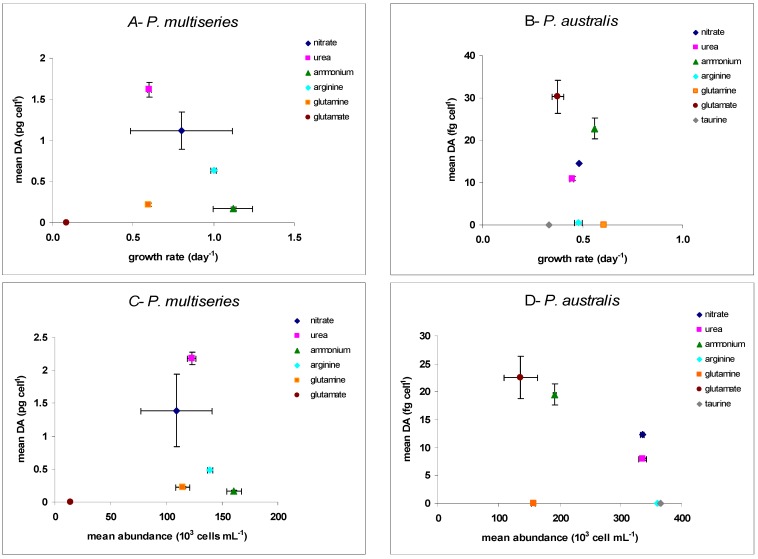
(**A**,**B**): mean cellular DA (average DA content measured during the exponential and stationary phases) as a function of specific growth rate (μ); (**C**,**D**): mean cellular DA in the stationary phase as a function of mean cell abundance in the stationary phase; (**A**,**C**) = *P. multiseries* CCL70 grown on nitrate, ammonium, urea, arginine, glutamine and glutamate; (**B**,**D**) = *P. australis* PNC1 grown on nitrate, ammonium, urea, arginine, glutamine, glutamate and taurine; *n* = 2 ± SE.

In both species, there was an interaction between the nitrogen source and the phases of growth, which was reflected in the cellular DA (Kruskall-Wallis test, *p* < 0.001). However, the two species exhibited different tendencies when comparing DA content to specific growth rate (Figure 3A,B) and no significant correlation was found (*P. multiseries*: *R*^2^ = 0.42; *P. australis*: *R*^2^ = 0.02). In contrast, both species showed an inverse relationship between the mean cellular DA content and the mean biomass produced during the stationary phase (Figure 3C,D). Correlation was not significant in *P. multiseries* (*R*^2^ = 0.387), while there was a significant linear negative correlation between cellular DA content and cell biomass (*y* = 9.10^−5^*x* + 40.8; *R*^2^ = 0.94) in cultures of *P. australis*. This correlation results from wide range of biomasses (cells mL^−1^) and narrow range of toxin levels (DA mL^−1^) among the different N conditions.

## 3. Discussion

### 3.1. Growth and Biomass

#### 3.1.1. Nitrate, Ammonium, Urea

The growth rates measured for *P. multiseries* strain CCL70 are within the range of those found in the literature for strains growing on nitrate and other nitrogen sources (Table 1, Table S2) [25,28,30,33,39,40,41,42,43,44,45]. No substantial differences in growth were obtained in our study for nitrate and urea, as already observed by Calu *et al.* [33] on the same strain, *P. multiseries* CCL70 (Table 1, Table S2). On the other hand, our strain exhibited growth responses different from strains used in other studies in which either nitrate, ammonium or urea were compared. The effect of inorganic nitrogen varied depending on the species and strain, but nitrate was usually the better inorganic nitrogen source for growth, sustaining higher growth rates and/or biomasses than ammonium [10,25,30] (Table S2). Bates *et al.* [25] obtained with *P. multiseries* strains TKA-2, MD-1 and NPARL a significantly higher cell yield in cultures supplied with nitrate than with ammonium, at 440 μM-N (Table S2). Their strains appeared sensitive to high concentrations of ammonium, which even induced growth inhibition when supplied at 880 μM [25] (Table S2). *P. multiseries* strain KP 84 also appeared sensitive to ammonium (300 μM), while higher growth rates were sustained with both nitrate and organic sources [30] (Table S2). In contrast, ammonium supported the best growth in our study. The biomass of our strain CCL70 was not affected by high concentrations of ammonium, which enabled the highest growth rate and an almost doubled cell yield compared to nitrate, for a 440 μM-N initial concentration (Table 1). Similarly, Thessen *et al.* [28] measured a high growth rate in strains CLN-47 and Pn-1 with ammonium provided at 88 μM, equivalent to that obtained with nitrate (Table S2). Ammonium is generally considered as preferentially assimilated compared to nitrate due to its low energy requirement [2,46]. However, it is toxic when provided at high levels, and Bates *et al.* [25] interpreted growth inhibition as the result of ammonium toxicity when the nitrogen concentration exceeded 200 μM. For strains TKA-2, MD-1 and NPARL, only ammonium concentrations of 55 to 110 μM allowed growth rates that were within the range of those obtained with nitrate [25] (Table S2). Indeed, the high growth rates measured by Thessen *et al.* [28] for strains CLN-47 and Pn-1 were obtained for an ammonium concentration (88 μM) greatly below that used in our study (Table S2). The negative effect of ammonium on the growth of *P. multiseries* KP 84 also started at a low concentration (20 μM) [30]. Interestingly, our strain of *P. multiseries* benefitted from high concentrations of ammonium above the 200 μM threshold determined by Bates *et al.* [25] to produce high biomass. The statement that growth inhibition is regulated by the ammonium concentration [25] may be thus strain-dependent. In comparison with previous studies, the efficient use of a high concentration (440 μM) of ammonium as a nitrogen source appears characteristic of the *P. multiseries* strain CCL70. Nevertheless, it is likely that strain CCL70 was affected by ammonium because *P. multiseries* exhibited a lag of several days before growth began in the presence of ammonium compared to other nitrogen treatments (Figure 1). In contrast, urea appeared to be a suitable nutrient for growth, sustaining a high biomass of our strain CCL70 and a high growth rate of strains CLN-47 [28] and KP 84 [30] (Table 1, Table S2).

Few studies addressed the growth and DA production of *P. australis* with respect to nitrogen sources, and most used nitrate as the nitrogen substrate [31,47,48,49,50,51,52] (Table S2). Howard *et al.* [31] compared strain AU221-a growing on nitrate, ammonium and urea. Specific growth rates of our strain PNC1 were slightly lower (0.33 to 0.60 day^−1^), but still in the same range as in their study (0.52 to 0.93 day^−1^) and other data (Table 1, Table S2). Howard *et al.* [31] obtained a twofold higher growth rate and a significantly higher biomass with ammonium and nitrate than with urea. Cochlan *et al.* also showed a lower affinity of *P. australis* for urea was [32] for strain AU-03184-5D. These authors demonstrated that uptake can vary depending on the nitrogen species, leading to a preference for nitrate, followed by glutamine and ammonium, which were taken up equally. In contrast, no substantial difference in growth rate was found in our study among these three nitrogen conditions (Table 1). Our strain PNC1, however, used urea as effectively as nitrate to produce a high biomass, while ammonium resulted in a lower cell abundance than the other two nitrogen sources (Table 1). Just as *P. multiseries* showed a growth sensitivity to high concentration of ammonium [25], we cannot exclude such sensitivity with our strain of *P. australis*; indeed Howard *et al.* [31] used enrichments of 50 μM-N, whereas we tested our strain with 400 μM-N.

Our study confirms the diversity of nutritional patterns with respect to ammonium and nitrate in diatoms [53,54,55,56,57] and already observed in several *Pseudo-nitzschia* species in culture. Nitrate and ammonium supported growth equally for most of the species previously studied, e.g., *P. cuspidata* [29], *P. delicatissima* [42] and one strain of *P. calliantha* [30]. On the other hand, another strain of *P. calliantha* was shown to grow better with ammonium, and *P. fraudulenta* grew faster on either nitrate or ammonium, depending on the strain [28]. The use of urea as a nitrogen source has been documented in only a few studies; these mostly showed that *Pseudo-nitzschia* can achieve similar growth rates on urea compared to nitrate and ammonium [28,30,31,33] (Table S2). In the same way, urea sustained a good growth rate and high cell yield of our *P. multiseries* and *P. australis* strains. The nutritional behavior we observed in two European strains of *P. multiseries* and *P. australis* thus supports the inter- and intra-specific variability in the physiology of *Pseudo-nitzschia* for nitrate, ammonium and urea, as previously stated by Lelong *et al.* [10] and Thessen *et al.* [28] for other *Pseudo-nitzschia* species.

#### 3.1.2. Amino Acids

There are few studies on the effect of organic nitrogen sources other than urea on the growth and toxin production of *Pseudo-nitzschia*, and of *P. multiseries* and *P. australis* in particular (Table S2). To our knowledge, arginine and taurine have never been studied as single sources in cultures of *Pseudo-nitzschia*. Glutamate was added to cultures of *P. multiseries* KP-59 but, because of the presence of nitrate, it was difficult to interpret the effects of this amino acid on growth [58,59]. Glutamine has been studied with respect to the growth of *P. multiseries* [30] and the uptake kinetics of *P. australis* [32]. Furthermore, although glutamate and glutamine have been shown to sustain dark survival of *P. multiseries* [60], the ability of *Pseudo-nitzschia* to grow on these amino acids is still debated. In our study, despite an overall ability to grow with all tested amino acids, *P. multiseries* CCL70 and *P. australis* PNC1 reacted differently to each of them. However, the same short lag phase observed for each growth curve with amino acids, urea or nitrate treatment (Figure 1 and Figure 2) indicates that the different uptake systems were equally induced under all nitrogen conditions, for both species. Responses of our species may thus be the result of a differential metabolic capacity towards the different amino acids that sustained growth. *P. multiseries* did not show preferential growth with organic nitrogen compared to inorganic nitrogen sources. Likewise, *P. australis* was able to grow with all nitrogen sources.

Taurine in particular produced a high biomass for our *P. australis* strains. This amino acid is generally known as a source of sulfur for growth [3], but its use as a nitrogen source has been demonstrated in the diatoms *Nitzschia* sp. cf. *ovalis* and *N. acicularis* [37]. It is noteworthy that the growth rate and maximum abundance of *P. australis* were similar in cultures that produced DA and in those that did not (growth on glutamine, arginine and taurine). Arginine always supported the highest cell yield and glutamate was the least effective in supporting the growth of both species. Uptake of arginine has been described in pennate diatoms [3,61,62]. Interestingly, the ability of our two *Pseudo-nitzschia* species to grow on this amino acid indicates that it serves as a nitrogen source for biomass production. Furthermore, the cell yield was equivalent with growth on arginine and on nitrate, as well as on urea for *P. australis*, or on ammonium for *P. multiseries*. The urea cycle was recently shown to be fully integrated into diatom metabolism [63,64]. During growth of our two species, arginine may thus have been catabolized by arginase or agmatinase to produce urea [64]. This nitrogen form may have been used further by our two *Pseudo-nitzschia* species, or in the form of ammonium via the urease activity, which catalyzed urea degradation into ammonia and carbonic acid.

Glutamate was used for growth by our *P. australis* strain but it did not support a biomass as high as did arginine and taurine. It is, however, notable that a similar biomass was achieved in our strain growing with glutamate or glutamine. Not all microalgae use glutamate, and growth is sometimes poorly supported by this amino acid, but particularly in centric diatoms [65,66]. On the other hand, glutamate assimilation has been shown in 18 species of pennate diatoms belonging to the genera *Navicula*, *Nitzschia*, *Amphiprora*, *Fragilaria*, *Amphora*, *Pleurosigma* and *Phaeodactylum*. This represents 75% of the tested strains [3,61,65,66,67,68,69]. It is thus notable that most of the pennate diatoms studied previously are able to use glutamate, even as the sole nitrogen source. Similarly, this amino acid sustained the growth of *P. multiseries*, as shown by Lyons [42] with strain CLN-1 (Table S2). In contrast, glutamate was not a good substrate for our *P. multiseries* strain CCL70, although this species grew well with glutamine (Table 1). This indicates that regulation of glutamine assimilation in CCL70 is different from that of glutamate. Differences in the ability to assimilate glutamate or glutamine have not been fully explained, even though these two amino acids are closely interconnected within the GS/GOGAT enzymatic system [2]. As well, both participate in the initial steps of nitrogen fixation in the cell, after which nitrogen is transferred to other amino acids by transamination [2]. In general, glutamine is a good source of nitrogen for algal growth [2]. Indeed Hillebrand and Sommer [30] showed that this amino acid was the best growth substrate for *P. multiseries* strain KP 84 (Table S2). In an extensive survey, Neilson and Larsson [67] reported that glutamine was widely used as a nitrogen source, while glutamate supported the growth of fewer than half of the species that grew with glutamine. The difference between the use of glutamine *vs.* glutamate was shown to be due to the impermeability of the cell wall to glutamate [3]. Such a difference in uptake ability has been observed in *Chlorella,* where glutamate uptake was seen to be strain-dependent [67,70]. Even though glutamate impermeability seems to be relatively rare [3], inability of our *P. multiseries* strain to grow on this substrate may be explained by a low ability of this species to take up the amino acid, while our *P. australis* strain maintained this function and was capable of growing with glutamate.

No simple rule has thus emerged governing the capacity of our *Pseudo-nitzschia* strains to utilize a given organic nitrogen source. Amino acids are known to be potentially taken up as valuable N-sources to supply all the nitrogen required for growth [3]. However, it is now known that the growth response is mostly related to the ability of each species and strain to take up each amino acid from the medium and to regulate the first step of nitrogen assimilation [3]. This is partly the result of specific transporters that determine the uptake of acidic, basic or neutral amino acids [3]. As in our study, in all previous studies on *Pseudo-nitzschia*, but as well as other diatoms, the ability to use organic N appears to be a species trait rather than a general nutritional type. This diversity of nutritional behavior, even within the same genus or species, has been described in the microalgae literature [24,67]. The metabolic capacity of each strain appears to be an outcome of selective pressure on a population subjected to different organic sources. It is also likely that strains lose their original metabolic capacity when maintained in culture over many years [10]. Based on a diatom’s genome [71], the selective maintenance or deletion of genes during the evolution of a group or species should determine the nutritional characteristics of each strain. The broad geographical distribution of the genus *Pseudo-nitzschia* [7,10] may explain its genotypic as well as phenotypic variability and why this species shows such a wide variability in growth characteristics and diverse behavior patterns towards inorganic and organic nitrogen sources.

### 3.2. Toxicity

#### 3.2.1. *P. multiseries* and *P. australis*

The cellular DA content of *P. multiseries* CCL70 (maximum 2.03 pg DA cell^−1^ with nitrate and 2.53 pg DA cell^−1^ with urea (Table S1) is consistent with that presented by Calu *et al.* [33] (maximum 3.16 pg DA cell^−1^ with nitrate and 5.17 pg DA cell^−1^ with urea) (Table S2), who used the same strain. It is also in the range of previous results obtained with nitrate, urea or ammonium substrates in other *P. multiseries* strains (Table S2). The DA contents are, however, among the lower values given for this species by Trainer *et al.* (see Table 3 in [7]), which range from 0.006 to 67 pg DA cell^−1^. The nutritional patterns of strain CCL70 were similar to those of strains Pn-1 and CLN-47, which produced the highest cellular DA while growing on either urea or nitrate [28] (Table 1, Table S2). In contrast, Bates *et al.* [25] reported a DA content two to four times higher with ammonium than with nitrate for strain NPARL grown with 220 μM-N and 440 μM-N (Table S2). As already noted for growth, these authors interpreted the high DA production as the physiological stress of cells due to a high concentration of ammonium in the medium. This was supported in their study by similar DA levels for both nitrate and ammonium at lower, non-toxic, concentrations (110 and 55 μM-N, Table S2). In contrast, in our experiment, ammonium supported the lowest cellular DA (mean value 0.17 pg·cell^−1^) in comparison with other nitrogen sources (Table 1). Nevertheless, the toxin production by our strain CCL70 occurred during the lag and exponential phases, whereas toxicity appeared only in the stationary phase with all other nitrogen substrates. This particular pattern of DA production of our strain indicates a metabolic shift of the cell population in the ammonium treatment that may have resulted from a physiological stress in the presence of high concentration of ammonium; this was illustrated by a long lag phase, as already observed for another *P. multiseries* strain, NPARL [25]. In contrast, the low DA content we measured in *P. multiseries* CCL70 with the glutamine treatment, although similar to that obtained with ammonium (Table 1), resulted from a different metabolic regulation. In the case of glutamine, DA was present in stationary-phase cells (Figure 1). This pattern is similar to that observed in most previous studies, which considered that when cell division slowed or ceased, cell metabolism was diverted to toxin production. Conversely, glutamate did not promote DA production by our *P. multiseries* CCL70 (Table 1). Another strain (CLN-1) grew and produced 34 times more DA in the culture supplied with 28 mM-GLU than in the control [42]. However, in the same study, strain CL-125 did not produce detectable DA when grown with this amino acid [42]. Thus, it seems that, as for the inorganic nitrogen sources, the intraspecific variability in physiology toward glutamate concerns not only growth but also toxin production by *Pseudo-nitzschia*. With regard to other amino acids, to our knowledge, the present study is the first to demonstrate toxin production by *P. multiseries* growing on arginine and glutamine (Table S2), although no toxin was produced by *P. australis* with these substrates.

The maximum DA concentrations appear highly variable among strains of *P. australis*, from 0.26 fg to 37 pg DA cell^−1^ [7] (Table S2). The cellular DA of our *P. australis* strain PNC1 was very low (10.8 to 30.3 fg·cell^−1^), close to values measured in *P. calliantha* and *P. fraudulenta* by Thessen *et al.* [28] and lower than most of the cellular DA values determined in other *P. australis* strains (Table 1, Table S2). In particular, Garrison *et al.* [47] reported high levels of DA for the strains DOMA-1 and DOMA-2 grown with nitrate (12 and 37 pg DA cell^−1^, respectively) a concentration in three orders of magnitude higher than in our *P. australis* strain (Table 1, Table S2). Nevertheless, the low DA concentration of our strain PNC1 cannot result from a loss of ability to produce toxin, often observed when cultures are maintained in the laboratory, because this strain was isolated during the year of the experiments. In contrast, cellular DA in our PNC1 strain is well above those found by Howard *et al.* [31] in strain AU221-a (Table S2). Furthermore, the nutritional patterns of each of these strains are different. Higher DA levels were measured with urea than with nitrate and ammonium substrates in their study, whereas the pattern was exactly the opposite in our experiments. It is notable that our *P. australis* produced high levels of DA while growing with glutamate. However, this amino acid supported a lower biomass than the other nitrogen sources that induced toxicity, particularly nitrate and urea (Table 1, Figure 2). Although arginine, glutamine and taurine are known to be good substrates for microalgae [3] and sustained the growth of *P. australis* PNC1, they did not induce DA production in our strain. In our study, neither cell leakage nor accumulation of DA in the medium was observed in *P. australis* grown with these three amino acids (Table S1). Release of DA into the medium can thus not explain our results, in contrast to the observations of Thessen *et al.* [28], who detected DA in the dissolved but not the particulate fraction of *P. fraudulenta* Pn-9 grown on ammonium and urea.

#### 3.2.2. Timing of DA Production

The timing of DA production was highly variable in our cultures, depending on both the *Pseudo-nitzschia* species and the nitrogen source (Figure 1 and Figure 2). Nevertheless, the highest DA content was measured for both species during the stationary phase in most of the nitrogen conditions tested, but the timing varied from early- to late-stationary phase. In accordance with the literature [10,11,20], DA production was associated with the slowing or cessation of cell division, most likely resulting from nutrient limitation. Similarly, toxin has been detected in nutrient-limited populations of *P. multiseries*, *P. seriata*, *P. fraudulenta*, *P. calliantha* and *P. australis* [20,25,26,28,41,72,73]. On the other hand, we also quantified cellular DA in fast-growing populations in the exponential phase, for both our species. Because the inocula of all nitrogen treatments were provided from the same initial stock culture, early DA production in some treatments could be interpreted as a specific consequence of the nitrogen source provided. Factors that triggered toxin production were, however, probably not identical for our two species. In *P. multiseries* cultures, only the ammonium treatment led to toxin production in the lag and early exponential phases. All other nitrogen sources initiated toxicity of *P. multiseries* later in the growth cycle, during the early- to mid-stationary phase. Because our inoculum tested negative for DA, the presence of DA during early exponential phase of the ammonium-grown cultures could not be explained by the statement of Thessen *et al.* [28], who considered that the DA detected before the stationary phase in their cultures of *P. multiseries*, *P. fraudulenta* and *P. calliantha* was caused by the carry-over of DA in the inoculum they used. It is thus likely that DA production in the ammonium treatment was an artifact of stressed cells, as previously described for other strains of *P. multiseries* [25].

On the other hand, DA production by *P. australis* grown with each nitrogen source started in the mid- to late-exponential phase. An exception was DA measured in the early exponential phase in the ammonium treatment, perhaps as a result of cell physiological stress in response to ammonium toxicity, as this was already observed for *P. multiseries* ([19], this study). However, an increase in DA production was observed during the stationary phase in this treatment, in contrast to the short period of DA production in *P. multiseries. P. australis* cellular DA measured later in all other treatments in mid-exponential phase, is likely not an artifact due to toxicity of the nitrogen source. It is noteworthy that Garrison *et al.* [47] also reported DA production by one strain of *P. australis* during the exponential phase, while growing on nitrate. Similarly, *P. australis* WW4 grown on nitrate produced DA during the late-exponential phase, as well as in the stationary phase [48]. Howard *et al.* [31] measured DA during the late-exponential growth phase in *P. australis* AU221 growing on nitrate, ammonium and urea. In contrast to *P. multiseries*, the ability to produce DA during the exponential phase thus seems a usual pattern in *P. australis*. Interestingly, this early toxin production during the exponential phase has also been documented in *P. cuspidata* [29] and *P.* sp. cf. *pseudodelicatissima* [74] and appears as a common trend in many *Pseudo-nitzschia* species.

#### 3.2.3. DA Production with Respect to Growth and Biomass

DA is a secondary metabolite and its production is generally considered to result from decreasing growth activity. It was suggested in this case that cells with low division rate or unable to divide are still able to photosynthesize and carry out secondary metabolism, including toxin production [10,73,75,76,77]. The ability of cells to synthesize DA thus seems inversely related to their primary metabolic activity during the period of toxin production [73,75,76,77]. On the other hand, regulation of DA production by energy and precursors accumulated during active growth has also been suggested, such that high toxin production can also be expected in relation to high growth rate [75,78]. In this case, one might expect a positive relationship between specific growth rate and DA production in batch culture. In our study, however, the highest DA content for both species was related to neither the highest nor the lowest growth rate. Specific growth rates and toxin production did not track each other, although there was an inverse but non-significant correlation between the specific growth rate and the mean DA content for our species. Few studies have examined the relationships between primary and secondary metabolisms in batch culture and they did not consider a broad range of nitrogen substrates. Thessen *et al.* [28] compared the effects of organic and inorganic forms using nitrate, ammonium and urea, and they did not find a significant relationship between growth rate and DA production in 17 of the 19 strains studied. Only one strain of *P. multiseries* (Pn-1) showed a positive correlation between growth rate and DA content, while an inverse relationship between the two parameters was reported for one strain of *P. fraudulenta* (Pn-9) [28]. Likewise, an inverse trend was found by Howard *et al.* [31] between cellular DA and growth rate of *P. australis* AU221-a growing in different nitrogen sources (nitrate, ammonium, urea). Given that all these studies showed that nitrogen substrates could affect growth and DA content differently, the regulation of *Pseudo-nitzschia* toxicity appears highly complex. Likewise, Auro [29] could not determine a clear relationship between the growth phase and DA production for *P. cuspidata* growing on nitrate, ammonium and urea. The cells’ ability to use nitrogen efficiently for primary metabolism (*i.e.*, as indicated by high growth rates) does not seem to influence directly their secondary metabolism, such that differences in specific growth rate in response to the nitrogen source are not reflected in toxin production.

Conversely, it could be argued that the best substrate to produce a high biomass also induces the highest production of toxin [78]. In contrast, our results showed that good growth, as defined by high cell yield, did not induce a greater potential for the cells to produce toxin. This is supported by the significant negative linear correlation between DA content and cell biomass in stationary phase for our *P. australis* strain. However, this was not the case for our *P. multiseries* strain, suggesting that there is no general nutritional rule governing the regulation of primary and secondary metabolism with respect to DA production. Even if DA as a secondary metabolite is not directly correlated to growth, the mechanism that allows cells to produce toxin may still be dependent on their ability to sustain the primary metabolism and divide to produce new biomass. Indeed, the findings that some amino acids, e.g., glutamine, arginine and taurine, supported growth of *P. australis* without producing DA reveals that, although DA contains nitrogen, its biosynthesis is not directly dependent on nitrogen availability. DA production may thus reflect specific characteristics of each nitrogen substrate for each *Pseudo-nitzschia* species. Previous work, using nitrate, ammonium and urea, failed to show such a discrepancy among these three nitrogen sources [28]. Hence, our results suggest that the various chemical forms of organic and inorganic nitrogen affect DA production not only by their direct effect on growth, but also by regulating resources between primary and secondary metabolic pathways during all stages of growth.

#### 3.2.4. DA Production with Respect to Nitrogen Sources and Energetics

Previous studies demonstrated that DA production occurred under unfavourable growth conditions when major nutrients such as phosphate or silicate were growth-limiting [7,10,11,20]. This was shown in particular for *P. multiseries* [43,73,77,79]. It was assumed in these cases that *Pseudo-nitzschia* cells with a low capacity to divide still provided sufficient energy by diverting resources toward toxin synthesis [75,76]. DA synthesis under excess nitrogen availability is regulated differently than when other elements are limiting, because nitrogen is required for the DA molecule [75]. Ammonium is a preferred nitrogen source for algae, as it is the most energetically efficient, in contrast to nitrate that requires large amounts of energy and reductants from photosynthesis for its assimilation [2,3,46]. DA production was, however, higher when *P. multiseries* cells were grown with nitrate than with ammonium, which is contrary to the energetic hypothesis. The inability of *P. australis* to produce DA when grown with glutamine or arginine, while these two amino acids allowed toxicity in *P. multiseries*, also contradicts the assumption of a direct control of DA metabolism by the nutritional conditions. Furthermore, while the uptake of inorganic nitrogen and urea may be coupled to photosynthesis, uptake rates of amino acids are not directly correlated with photosynthesis [80]. In particular, regulation of the uptake of basic amino acids is different from that of other nitrogen sources [80]. The higher DA production in *P. multiseries* grown with nitrate and urea than with the amino acids therefore appears not to be directly related to the energetic status of the cells which would depend only on photosynthesis. Conversely, in *P. australis*, toxin synthesis was highest with glutamate, which has a metabolism close to that of glutamine; the latter, however, sustained a similar growth yield, but without toxicity. In this particular case, it is possible that the higher DA production while using glutamate resulted from a lower energetic demand for this amino acid compared to glutamine, because glutamate is considered a precursor of DA in its biosynthetic pathway [81]. Moreover, whereas amino acids are generally considered a nitrogen source, glutamate is also known to provide carbon. Thus, this amino acid has often been shown to be used by microalgae to sustain carbon anabolism [3]. It thus appears that, despite the control that each nitrogen source may exert, no simple rule related to the energy requirement has emerged governing the capacity of our two species to use a given substrate. The preferential channelling of carbon toward DA synthesis seems not to be determined by each nitrogen source, as one would expect. The variability in DA production for the same nitrogen source suggests that conditions favouring DA synthesis are not directly linked to the energy requirement for assimilating each nitrogen species. Rather the metabolic requirement of each strain in our study seems to have determined the amount of carbon and energy to DA production.

In the end, it is noteworthy that, while both of our *Pseudo-nitzschia* species showed good growth, their expression of DA biosynthesis always depended on the nitrogen substrate. One could hypothesize that because DA is a nitrogenous molecule, any of the nitrogen substrates would exert a similar control on growth and toxin production. Yet, in accordance with previous studies [28,29], but now considering an even broader range of nitrogen sources, it seems that there is no common mechanism for explaining DA production as a function of different nitrogen sources. Rather each *Pseudo-nitzschia* strain possesses its own metabolic capacity and physiological approach for nitrogen assimilation, which could explain differences in toxicity. What is new is that this is just as complex for organic as for inorganic nitrogen sources. It is, however, not clear to what degree our results can be attributed to inter- and/or intra-specific variability in the physiology of *Pseudo-nitzschia*; the diversity of responses requires future studies to discern the mechanisms regulating growth *vs.* DA production. Recent information on the *Pseudo-nitzschia* genome [82] will greatly aid further understanding of its metabolism.

## 4. Experimental Section

### 4.1. Cell Cultures

*P. multiseries* strain CCL70 was isolated in 2007, from the Thames estuary (UK) by CEFAS (Center for Environment, Fisheries and Aquaculture Science). *P. australis* strain PNC1 was isolated from the Bay of Crozon (France Atlantic coast) in January 2010, by Ifremer (Institut Français de Recherche pour l’Exploitation de la Mer). Toxicity of both strains was previously confirmed by DA analysis by Liquid Chromatography coupled with tandem Mass Spectrometry (LC/MS-MS). 

The two species were grown in sterile-filtered (0.2 μm) natural seawater (English Channel) enriched with medium L1 nutrients (N-NO_3_ source) + 107 µM silicate [83]. Stock cultures were maintained in 225 mL sterile polystyrene cell culture flasks with vented caps (Iwaki, ASAHI GLASS CO., LTD., Japan) in a temperature-controlled chamber at 16 °C, under a 12:12 h light:dark cycle. Irradiance was provided by daylight fluorescent tubes at an average level of 110 µmol photons m^−2^·s^−1^. Because the two species were not axenic, stock cultures were periodically checked for bacterial development and treated (1 mL·L^−1^ culture medium) with an antibiotic mixture (SIGMA A5955: penicilline (5 g·l^−1^) + streptomycine (5 g·l^−1^) + neomycine 10 g·l^−1^) to ensure minimal bacterial contamination.

### 4.2. Experimental Procedures

For the nitrogen experiments, the L1 culture medium was prepared without any added nitrogen. Seawater was analyzed for major nutrients. Initial concentrations were 0.49 μM and 6.8 μM for ammonium and silicate, respectively, and were below the analytical threshold for nitrate and urea. To ensure uniformity for all nitrogen sources, nitrogen enrichments were calculated as a molar equivalence of 440 μM-N. Tests were performed using two inorganic (nitrate and ammonium) and five organic (urea, arginine, glutamine, glutamate and taurine) substrates. Experiments were carried using duplicate cultures of each nitrogen condition, plus a control treatment without added nitrogen.

*Pseudo-nitzschia* is a delicate microalgae that can die rapidly or lose its toxicity when maintained in culture [10]. To avoid any changes in physiology during a long maintenance period in different nitrogen sources, we chose not to acclimatize the two species to the different nitrogen sources before the study. Rather we tested the same nitrogen-starved inoculum. This allowed us to compare each species using cells of similar physiological status. Before the nitrogen experiments, *Pseudo-nitzschia* cells from stock cultures previously grown with nitrate as the nitrogen source were first starved for seven days in seawater medium (L1 + Si) with no added nitrogen under the above conditions. After starvation, 5 mL of *P. multiseries* or *P. australis* were inoculated into sterile polystyrene 225-mL flasks with vented caps (Iwaki) containing 200 mL of the different nitrogen-enriched media, to achieve an initial concentration of 5700 cells mL^−1^. The absence of detectable DA in the inoculum was verified before the nitrogen enrichment. Experimental batch cultures were then maintained a temperature-controlled chamber as described above. Growth was monitored for three weeks. Experimental cultures were stirred and sampled daily for cell biomass and toxin measurements.

### 4.3. Cell Quantification

Cell samples were fixed in 1% acidic Lugol’s solution. *Pseudo-nitzschia* cells were enumerated under a light microscope using a Nageotte or Mallassez cell chamber. The precision of counts was estimated following Lund *et al.* [84]. The confidence interval was *p* < 0.10. Growth rates were calculated as μ = ln (*X*_2_) − ln (*X*_1_)/*t*_2_ − *t*_1_, where *X* = cell abundance (cells mL^−1^) and *t* = time (day).

### 4.4. Domoic Acid Analysis

For DA extraction, 10 mL of culture was centrifuged for 20 min at 3600× *g*. The supernatant was collected for dissolved DA and frozen at −80 °C. The cell pellet was placed in 50:50 *v*/*v* methanol:water and frozen at −80 °C until DA extraction. Intracellular DA was then recovered by sonicating the pellet (500 W, 20 kHz, 30 min). Samples of both dissolved and particulate DA were filtered onto a GF/C Whatman membrane under centrifugation (8000× *g*, 10 min) before HPLC injection. Intracellular and dissolved DA were analyzed separately according to the HPLC-UV method established by Amzil *et al.* [85], based on the method of Quilliam *et al.* [86], using a C_18_ reverse-phase column (Jupiter; S: 5 μm; 4.6 mm× 250 mm) thermostatically controlled at 40 °C; mobile phase 90:10 H_2_O:0.1% trifluoracetic acid/acetonitrile at a flow rate of 1 mL·min^−1^. The injected volume was 20 µL. DA was detected by UV absorbance (λ = 242 nm). The DA in the sample was quantified in duplicate using a certified standard DA solution from the National Research Council of Canada (Halifax, Nova Scotia). Chromatograms were analyzed using the software Agilent ChemStation for LC 3D. The detection limit (signal/background = 3) was 22 ng DA mL^−1^.

### 4.5. Data Analysis

Data are expressed as mean ± standard deviation (SD). Statistical analyses consisted of multifactor analyses of variance (ANOVA) or non-parametric test (Kruskall Wallis). Differences were considered significant at *p* < 0.05. All statistical analyses were carried out using the software Statgraphics Centurion XV.I (StatPoint Technologies Inc., Warrenton, VA, USA).

## 5. Conclusions

Our results support the conclusions of previous studies regarding the broad differences in physiology among species and strains of *Pseudo-nitzschia*. No general rule could be demonstrated for differences in growth and toxicity as a function of several inorganic (nitrate, ammonium) and organic (urea, glutamate, glutamine, arginine, taurine) nitrogen substrates, for two representative toxigenic species, *P. multiseries* and *P. australis*. No specific variation in growth rate, cell abundance nor DA content could be attributed to either the inorganic or organic nitrogen substrates. Our *P. multiseries* strain produced a higher biomass and more cellular DA when grown with ammonium and urea, respectively. On the other hand, *P. australis* benefitted more from organic substrates and grew better with taurine, while producing the highest DA concentration with glutamate. Our results show that *Pseudo-nitzschia* can readily utilize organic nitrogen in the form of amino acids, while previous studies mainly focused on inorganic nitrogen. Organic nitrogen nutrition, however, did not always induce toxicity. Glutamine, arginine and taurine supported the growth of *P. australis*, but without DA production. In contrast, *P. multiseries* was able to grow and produce DA with both inorganic and organic substrates, with the exception of glutamate. This amino acid may not have been assimilated by our strain. The effect of the nitrogen source on growth and DA production thus appears unpredictable and highly variable, depending on the species. The present research, together with previous studies, does not provide evidence that one specific inorganic or organic nitrogen source is more conducive to sustaining growth or triggering toxicity in *Pseudo-nitzschia*. However, the general finding, previously reported for inorganic nutrients, that growth limitation induces toxicity was also observed for organic nitrogen sources. In accordance with the literature on different *Pseudo-nitzschia* species or strains, most toxin was produced during the stationary phase in most of our treatments, with the exception of ammonium. In other respects, it is noteworthy that more toxin was produced by cells with nitrogen sources that did not sustain a high biomass. A relationship thus seems to exist between the ability of *Pseudo-nitzschia* to grow and to produce toxin, which depends on the nature of the nitrogen source. This relationship is significant for *P. australis* in our study. The key is to determine the metabolic regulators that promote either cell biomass or DA production. Our understanding of the parameters playing a part in the biology of this diatom is complicated by the intra- and inter-specific physiological variability that characterizes the genus *Pseudo-nitzschia*. More robust conclusions could be drawn had several strains of each species or more species been studied. Nevertheless, our findings provide evidence that the overall regulation of *Pseudo-nitzschia* physiology is complex with respect to a broad range of inorganic and organic nitrogen sources. A better understanding of its nutritional physiology will help to identify bloom controls and to anticipate toxic events.

## Supplementary Files

Supplementary File 1
