# Supplementary material for: Effects of Organic and Inorganic Nitrogen on the Growth and Production of Domoic Acid by Pseudo-nitzschia multiseries and P. australis (Bacillariophyceae) in Culture
Source: Mar Drugs. 2015 Nov 26;13(12):7067–86. doi: 10.3390/md13127055 (PMC4699229; doi:10.3390/md13127055)
Supplement: Supplementary File 1 [file marinedrugs-13-07055-s001.pdf]

Supplementary Material

Table S1. Maximum cellular domoic acid (DA), mean cellular DA during the exponential and stationary phases, mean extracellular DA; for *P. multiseries* CCL70 and *P. australis* PNC1 grown on nitrate, ammonium, urea, arginine, glutamine, glutamate and taurine (the latter for *P. australis* only); nd = not detectable; $n = 2 \pm$ SE. ** We did not detect DA in the *P. multiseries* culture medium, in any of our treatments. It cannot be excluded that a methodological failure occurred during preservation of the vials containing dissolved DA, because extracellular DA has been reported in all previous studies on toxigenic *P. multiseries*.

	***P. multiseries* CCL70**			
	**Maximum DA (pg·Cell^{-1})**	**Mean DA Exponential Phase (pg·Cell^{-1})**	**Mean DA Stationary Phase (pg·Cell^{-1})**	**Mean DA Extracellular ****
Nitrate	2.03 ± 0.64	0.07 ± 0.04	1.39 ± 0.55	nd
Urea	2.53 ± 0.25	$0.03 \pm 3 \times 10^{-5}$	2.18 ± 0.09	nd
Ammonium	0.30 ± 0.04	0.24 ± 0.03	0.14 ± 0.01	nd
Arginine	0.87 ± 0.12	0.006 ± 0.01	0.49 ± 0.01	nd
Glutamine	0.60 ± 0.01	0.003 ± 0.005	0.22 ± 0.02	nd
Glutamate	nd	nd	nd	nd
	***P. australis* PNC1**			
	**Maximum DA (fg·Cell^{-1})**	**Mean DA Exponential Phase (fg·Cell^{-1})**	**Mean DA Stationary Phase (fg·Cell^{-1})**	**Mean DA Extracellular ng·mL^{-1}**
Nitrate	37.1 ± 12.7	2.4 ± 0.1	12.3 ± 3.3	6.7 ± 2.7
Urea	24.8 ± 5.9	2.3 ± 0.1	7.9 ± 0.2	15.5 ± 0.7
Ammonium	43.9 ± 2.3	13.4 ± 0.6	19.5 ± 1.9	15.4 ± 0.6
Arginine	nd	nd	nd	nd
Glutamine	nd	nd	nd	nd
Glutamate	79.0 ± 6.4	12.4 ± 0.2	22.6 ± 3.8	20.1
Taurine	nd	nd	nd	nd

Table S2. Specific growth rate, biomass in stationary phase and domoic acid (DA) content of *P. multiseries* and *P. australis* growing in batch culture with different nitrogen sources; GLN = glutamine; GLU = glutamate; na = not determined; nd = not detected.

Species	Strain (Origin)	N Source (μM-N)		Irradiance (μmol Photons $m^{-2} \cdot s^{-1}$)	Specific Growth Rate (d^{-1})	Biomass in Stationary Phase (10^3 Cells mL^{-1})	DA Content ($Cell^{-1}$)	Reference
P. multiseries	NPARL (Canada)	NO_3	880 [a]	40		129	0.2–10 pg	Bates *et al.* (1989) [1]
			880 [a]	100		30–205	0.04–21 pg	
			880 [a]	140		36	0.2–0.9 pg	
			880 [a]	165		129	1.3–18 pg	
		NO_3	50	100		90	0.4 pg	Bates *et al.* (1991) [2]
			1000	100		256	9.6 pg	
		NO_3	55	100		100 [b]	0.30 pg [b]	Bates *et al.* (1993) [3]
			110	100		160 [b]	1 pg [b]	
			220	100		240 [b]	1.4 pg [b]	
			440	100		180 [b]	1.7 pg [b]	
			440	100		300 [c]	0.6–4.2 pg [c]	
		NH_4	55	100		100 [b]	0.3 pg [b]	
			110	100		160 [b]	1 pg [b]	
			220	100		180 [b]	3.7 pg [b]	
			440	100		130 [b]	5 pg [b]	
			440	100		30 [c]	0.6–8.5 pg [c]	
	TKA-2 (USA)	NO_3	880 [a]	100	0.65	150 [d]	2 pg [e]	Douglas & Bates (1992) [4]
		NO_3	220	100		166		Bates *et al.* (1993) [3]
			440	100		203		
			880	100		195		
		NH_4	220	100		123		
			440	100		30		
			880	100		1		
	MD-1 (USA)	NO_3	220	100		246		Bates *et al.* (1993) [3]
			440	100		261		
			880	100		265		
		NH_4	220	100		178		
			440	100		82		
			880	100		1		

Table S2. *Cont.*

	KP 84 (Canada)	NO_3	200	230	0.42–0.55	na	na	Hillebrand & Sommer (1996) [5]
			200	25	0.42–0.66		na	
		Urea	200	25	0.38–0.60		na	
		NH_4	300	230	nd		na	
			300	25	0.33–0.36		na	
		GLN	200	25	0.65–0.83		na	
	NPBIO (Canada)	NO_3	1,760 [f]	410	0.20–0.25	140–150	144–432 fg	Pan *et al.* (1996) [6]
	CLN-1 (Canada)	GLU	28.10^3	100		733	0.067 pg	Lyons (2002) [7]
	Mu411P (USA)	NO_3	880 [a]	100			0.1–15 pg	Kudela (2003) [8]
P. multiseries	CL-195 (Canada)	NO_3	880 [g]	100			0.7 pg [e]	Lundholm *et al.* (2004) [9]
	OKPm013-2 (Japan)	NO_3	880 [g]	100			1.15 pg [e]	Lundholm *et al.* (2004) [9]
		NO_3	880 [a]	200			1.9–140 pg	Trimborn *et al.* (2007) [10]
	CCL70 (UK)	NO_3	440		0.56		3.16 pg [e]	Calu *et al.* (2009) [11]
		Urea	440		0.67		5.17 pg [e]	
	CLN-47 (Canada)	NO_3	88	150/200	0.65		362 fg	Thessen *et al.* (2009) [12]
		NH_4	88	150/200	0.61		48 fg	
		Urea	88	150/200	0.68		24 fg	
	Pn-1 (USA)	NO_3	88	150/200	0.76		1250 fg	Thessen *et al.* (2009) [12]
		NH_4	88	150/200	0.76		406 fg	
		Urea	88	150/200	0.30		1807 fg	

Table S2. *Cont.*

	DOMA-1 (USA)	NO_3	54	22	0.8	20	12 pg [e]	Garrison *et al.* (1992) [13]
	DOMA-2 (USA)	NO_3	54	32	0.82	20	37 pg [e]	Garrison *et al.* (1992) [13]
	WW4 (Ireland)	NO_3	880 [a]	115	0.73–0.94	73–97	0.24–26 pg	Cuzak (2002) [14]
	PLY1St.19A (UK)	NO_3	880 [a]	100		172	0.15 pg	Fehling *et al.* (2004) [15]
	PLY1St.54B (UK)	NO_3	880 [a]	100		129	1.68 pg	Fehling *et al.* (2004) [15]
P. australis	AU221-a (USA)	NO_3	50	100	0.89		0.48 fg	Howard *et al* (2007) [16]
		NH_4	50	100	0.93		0.26 fg	
		Urea	50	100	0.52		1.37 fg	
	PA2 (Chile)	NO_3	880 [a]	100–140			0.04 pg	Àlvarez *et al.* (2009) [17]
	PA3 (Chile)	NO_3	880 [a]	100–140			0.05 pg	Àlvarez *et al.* (2009) [17]
	PA4 (Chile)	NO_3	880 [a]	100–140			1.74 pg	Àlvarez *et al.* (2009) [17]
	BTS-1 (Mexico)	NO_3	880 [g]	200			0.11–3 pg	Santiago *et al.* (2011) [18]
	PNaus45 (France)	NO_3	440 [h]	35–400	0.47–0.83		0.5–0.7 pg	Thorel *et al.* (2014) [19] [i]

[a] = F/2 medium; [b,c] = recalculated values from Figures 2 and 3, respectively (Bates *et al*., 1993) [3]; [d] = recalculated value from Figure 1A (Douglas and Bates, 1992) [4]; [e] = maximum value; [f] = F medium; [g] = L1 medium; [h] = K/2 medium; [i] = data from semi-continuous culture.

References

1. Bates, S.S.; Bird, C.J.; de Freitas, A.S.W.; R. Foxall; Gilgan, M.; Hanic, L.A.; Johnson, G.R.; McCulloch, A.W.; Odense, P.; Pocklington, R.; *et al.* Pennate diatom *Nitzschia pungens* as the Primary source of domoic acid, a toxin in shellfish from Eastern Prince Edward Island, Canada. *Can. J. Fish. Aquat. Sci.* **1989**, *46*, 1203–1215.
2. Bates, S.S.; de Freitas, A.S.W.; Milley, J.E.; Pocklington, R.; Quilliam, M.A.; Smith, J.C.; Worms, J. Controls on domoic acid production by the diatom *Nitzschia pungens* f. *multiseries* in culture: Nutrients and irradiance. *Can. J. Fish. Aquat. Sci.* **1991**, *48*, 1136–1144.
3. Bates, S.S.; Worms, J.; Smith, J.C. Effects of ammonium and nitrate on domoic acid production by *Pseudo-nitzschia pungens* in batch culture. *Can. J. Fish. Aquat. Sci.* **1993**, *50*, 1248–1254.
4. Douglas, D.J.; Bates, S.S. Production of domoic acid, a neurotoxic amino acid, by an axenic culture of the marine diatom *Nitzschia pungens* f. *multiseries* Hasle. *Can. J. Fish. Aquat. Sci.* **1992**, *49*, 85–90.
5. Hillebrand, H.; Sommer, U. Nitrogenous nutrition of the potentially toxic diatom *Pseudonitzschia pungens* f. *multiseries* Hasle. *J. Plankton Res.* **1996**, *18*, 295–301.

6. Pan, Y.; Subba Rao, D.V.; Mann, K.H.; Brown, R.G.; Pocklington, R. Effect of silicate limitation on production of domoic acid, a neurotoxin, by the diatom *Pseudo-nitzschia multiseries*. I. Batch culture studies. *Mar. Ecol. Prog. Ser.* **1996**, *131*, 225–233.
7. Lyons, D. Effect of Organic Enrichment on Growth and Domoic Acid Production by Axenic Cultures of the Pennate Diatom *Pseudo-nitzschia multiseries*. Bachelor's Thesis, Mount Allison University, Sackville, New Brunswick, NB, Canada, 2002.
8. Kudela, R.; Roberts, A.; Armstrong, M. Laboratory analyses of nutrient stress and toxin production in *Pseudo-nitzschia* spp. from Monterey Bay, California. In *Harmful Algae*; Steidinger, K.A., Landsberg, J.H., Tomas, C.R., Vargo, G.A., Eds.; Florida Fish and Wildlife Conservation Commission, Florida Institute of Oceanography, and Intergovernmental Oceanographic Commission of UNESCO: Paris, France, 2002; pp. 136–138.
9. Lundholm, N.; Hansen, P.J.; Koyaki, Y. Effect of pH on growth and domoic acid production by potentially toxic diatoms of the genera *Pseudo-nitzschia* and *Nitzschia*. *Mar. Ecol. Prog. Ser.* **2004**, *273*, 1–15.
10. Trimborn, S.; Lundholm, N.; Thoms, S.; Richter, K.; Krock, B.; Hansen, P.J.; Rost, B. Inorganic carbon acquisition in potentially toxic and non-toxic diatoms: The effect of pH-induced changes in seawater carbonate chemistry. *Physiol. Plant.* **2007**, *133*, 92–105.
11. Calu, G.; Martin-Jézéquel, V.; Lefaux, E.; Séchet, V.; Lassus, P.; Weigel, P.; Amzil, Z. The influence of nitrogen speciation on growth and toxicity *of Pseudo-nitzschia multiseries* and *P. pungens* in batch and continuous cultures. In *7th International Conference on Molluscan Shellfish Safety*; Lassus, P., Ed.; Ifremer: Nantes, France, 2009; pp. 157-162.
12. Thessen, A.E.; Bowers, H.A.; Stoecker, D.K. Intra- and interspecies differences in growth and toxicity of *Pseudo-nitzschia* while using different nitrogen sources. *Harmful Algae* **2009**, *8*, 792–810.
13. Garrison, D.L.; Conrad, S.M.; Eilers, P.P.; Waldron, E.M. Confirmation of domoic acid production by *Pseudonitzschia australis* (Bacillariophyceae) cultures. *J. Phycol.* **1992**, *28*, 604–607.
14. Cusack, C.K.; Bates, S.S.; Quillliam, M.A.; Patching, J.W.; Raine, R. Confirmation of domoic acid production by *Pseudo-nitzschia australis* (Bacillariophyceae) isolated from Irish waters. *J. Phycol.* **2002**, *38*, 1106–1112.
15. Fehling, J.; Green, D.H.; Davidson, K.; Bolch, C.J.; Bates, S.S. Domoic acid production by *Pseudo-nitzschia seriata* (Bacillariophyceae) in Scottish waters. *J. Phycol.* **2004**, *40*, 622–630.
16. Howard, M.D.A; Cochlan, W.P.; Ladizinsky, N.; Kudela, R.M. Nitrogenous preference of toxigenic *Pseudo-nitzschia australis* (Bacillariophyceae) from field and laboratory experiments. *Harmful Algae* **2007**, *6*, 206–217.
17. Álvarez, G; Uribe, E.; Quijano-Scheggia, S.; López-Rivera, A.; Mariño, C.; Blanco, J. Domoic acid production by *Pseudo-nitzschia australis* and *Pseudo-nitzschia calliantha* isolated from North Chile. *Harmful Algae* **2009**, *8*, 938–945.
18. Santiago-Morales, I.S; García-Mendoza, E.G. Growth and domoic acid content of *Pseudo-nitzschia australis* isolated from northwestern Baja California, Mexico, cultured under batch conditions at different temperatures and two Si:NO_3 ratios. *Harmful Algae* **2011**, *12*, 82–94.

19. Thorel, M.; Fauchot, J.; Morelle, J.; Raimbault, V.; le Roy, B.; Miossec, C.; Kientz-Bouchart, V.; Claquin, P. Interactive effects of irradiance and temperature on growth and domoic acid production of the toxic diatom *Pseudo-nitzschia australis* (Bacillariophyceae). *Harmful Algae* **2014**, *39*, 232–241.

© 2015 by the authors; licensee MDPI, Basel, Switzerland. This article is an open access article distributed under the terms and conditions of the Creative Commons Attribution license (http://creativecommons.org/licenses/by/4.0/).
